# Different doses of nalmefene combined with hydromorphone hydrochloride for postoperative analgesia after colorectal surgery: a randomized controlled study

**DOI:** 10.1186/s12893-023-02293-z

**Published:** 2024-01-02

**Authors:** Ye Wang, Lin Zhao, Meng Wu, Qi An, Qianqian Guo, Chunling Fan, Zhenggang Guo

**Affiliations:** Department of Anaesthesiology, Peking University Shougang Hospital, No.9 Jinyuanzhuang Rd, Shijingshan District, Beijing, 100144 China

**Keywords:** Nalmefene, Hydromorphone hydrochloride, Colorectal cancer, Postoperative analgesia

## Abstract

**Background:**

Hydromorphone hydrochloride has a satisfactory postoperative analgesic effect for patients with colorectal cancer but is accompanied by a relatively high incidence of adverse events. Low-doses of naloxone combined with opioids for patient-controlled analgesia can reduce the incidence of drug-related adverse events. Nalmefene is a more selective opioid receptor antagonist than naloxone. The aim of this study was to determine the impact of low-doses of nalmefene on the analgesic effect and incidence of adverse events of patients with hydromorphone patient-controlled analgesia (PCA) undergoing colorectal radical surgery.

**Methods:**

Ninety-nine patients undergoing elective laparoscopic or hand-assisted laparoscopic radical surgery under general anaesthesia were randomly divided into three groups. Group N1 received hydromorphone hydrochloride 0.15 mg/kg + nalmefene 2 µg/kg; Group N2 received hydromorphone hydrochloride 0.15 mg/kg + nalmefene 0.5 µg/kg; and the control group (Group C) received hydromorphone hydrochloride 0.15 mg/kg. All medications were diluted to 100 ml with normal saline. The primary outcome was pain intensity at 12 h after surgery; the secondary outcomes were the occurrence of nausea, vomiting and pruritus and the total analgesic consumption of the PCA pump at 1 h, 6 h, 12 h, 24 and 48 h after surgery.

**Results:**

The NRS scores of Group N1 (2 µg/kg) were significantly lower than those of Group C (P = 0.025), and no difference was found between group N2 and group C (P > 0.05). Among the three groups, the NRS scores of Group N1 (2 µg/kg) were significantly lower than those of Group C at 12 h (P = 0.01) and 48 h (P = 0.01) postoperatively. Compared with 12 h postoperatively, the NRS scores were lower at 24 h postoperatively in Group N1 and Group C (P < 0.05) and significantly lower at 48 h postoperatively in all three groups (P < 0.001). There was a significant difference in the incidence of pruritus among the three groups (P = 0.036).

**Conclusions:**

Nalmefene at a dosage of 2 µg/kg enhances the postoperative analgesic effect of hydromorphone hydrochloride and reduces the occurrence of postoperative pruritus.

**Trial Registration:**

The trial was registered with the Chinese Clinical Trial Registry (Registration number: ChiCTR2000033520, date: 03/06/2020).

## Introduction

Colorectal cancer is a common malignancy of the digestive system. With an increasingly high incidence in China, it has ranked second among all cancers in recent decades [1]. Surgery is an effective therapeutic option for colorectal cancer. However, most patients will experience moderate to severe pain after open and minimally invasive colorectal surgery, despite following the ERAS perioperative program. Effective postoperative analgesia can reduce patients’ pain and promote recovery after surgery [2, 3].

Opioids are the primary medications for managing pain in surgical patients. Hydromorphone hydrochloride is a semisynthetic mu opioid agonist. Studies have demonstrated that hydromorphone has the valuable advantages of rapid onset and strong analgesic efficacy for analgesia. However, it might have a higher incidence of nausea and pruritus than sufentanil [4, 5]. Recent studies have found that low-dose opioid receptor antagonists, such as naloxone, could enhance the analgesic effect of opioids and decrease the incidences of postoperative nausea and pruritus after surgery [6, 7]. As a higher selectivity µ-receptor antagonist, nalmefene has a longer half-life than naloxone. Preoperative single intravenous administration of low-doses of nalmefene can reduce the incidence of postoperative opioid-related adverse events [8]. However, few clinical studies have focused on the use of nalmefene combined with hydromorphone hydrochloride for postoperative analgesia.

We hypothesized that low-doses of nalmefene combined with hydromorphone hydrochloride for postoperative analgesia in colorectal cancer patients could enhance the analgesic effect and reduce the occurrence of opioid-related adverse events. Therefore, a prospective, randomized, double-blind, controlled study was designed and performed to evaluate the effects of low-doses of nalmefene on postoperative analgesia and adverse events of hydromorphone hydrochloride in patients with colorectal cancer.

## Methods

### Ethics approval and participation consent

This prospective, double-blind, randomized controlled study was approved by the Ethics Committee of Peking University Shougang Hospital, Beijing, China (Ethical number: IRB-AF-25-02) and registered with the Chinese Clinical Trial Registry (http://www.chictr.org.cn. Registry number: ChiCTR2000033520, date: 03/06/2020). Informed written consent was obtained from all participants before enrolment.

### Criteria for inclusion and exclusion

**Fig. 1:**
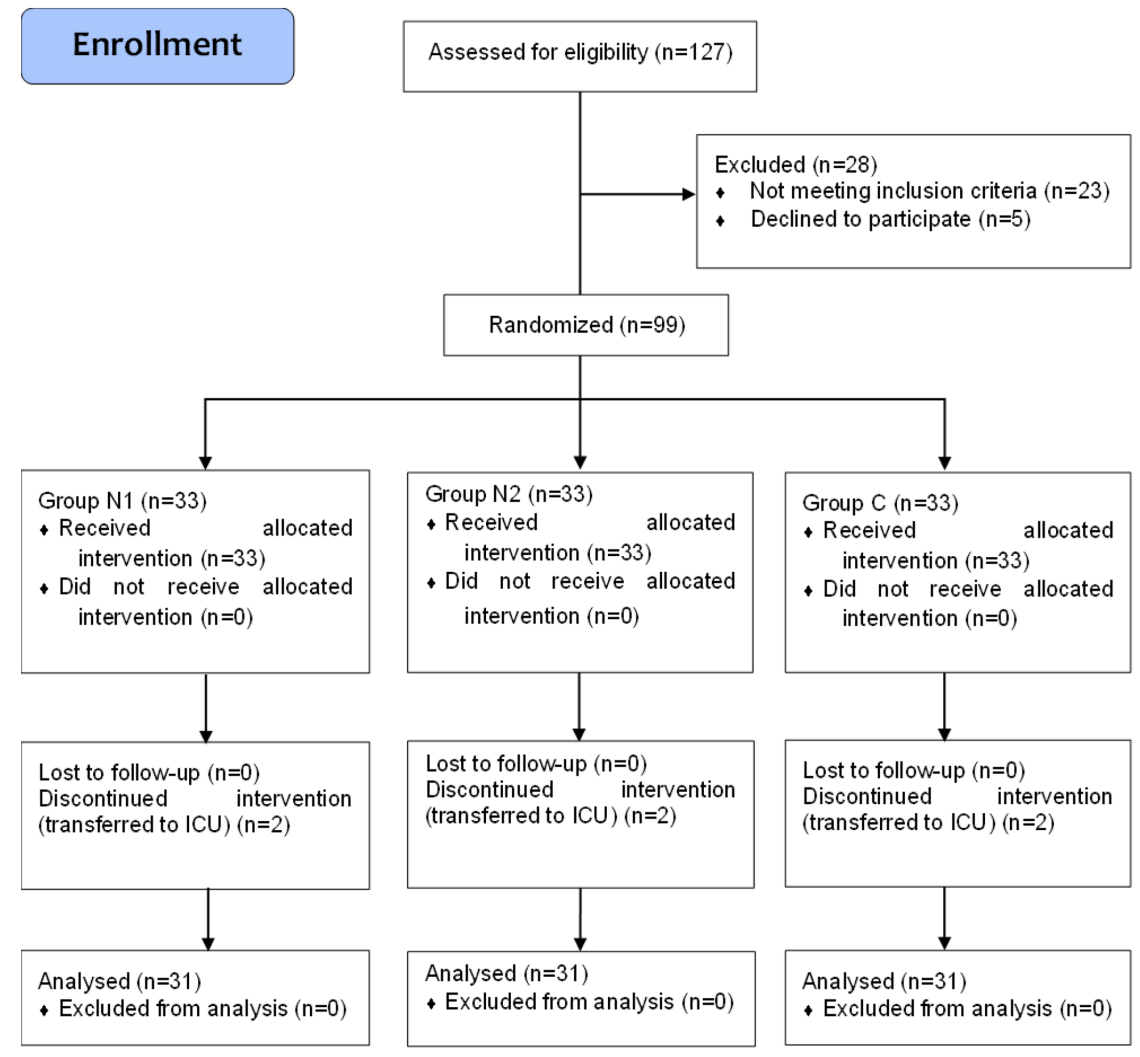
Consolidated Standards of Reporting Trials flow diagram

A total of 127 patients undergoing elective laparoscopic or hand-assisted laparoscopic colorectal radical surgery were recruited during June 2020 and September 2021 at Peking University Shougang Hospital. The inclusion criteria were as follows: aged 18–80 years; American Society of Anaesthesiologists (ASA) physical status I-III; BMI 18–30 kg/m^2^; voluntarily participated in the study; and signed informed consent forms. All patients were scheduled to receive general anaesthesia. The exclusion criteria were as follows: incision length > 10 cm; body mass index (BMI) ≤ 18 or ≥ 25 kg/m^2^; allergic to relevant drug; alcohol or opioid abuse; history of nausea, vomiting or skin disease; severe renal or hepatic dysfunction; neuropsychiatric disorder; inability to understand NRS or Ramsay Sedation Scale; inability to properly describe postoperative pain (e.g., language barrier) or inability to use IV-PCA pump; taking sedative, anti-emetic, or antipruritic drugs during the 24 h preoperatively; taking monoamine oxidase inhibitor (MAOI) or antidepressant use 15 days before surgery. The withdrawal criteria were as follows: transfer to the intensive care unit (ICU) unexpectedly postoperatively. The patient selection process is shown in Fig. 1.

### Randomization and blinding

Patients were randomly allocated into three groups according to an SPSS Statistics-generated randomization schedule using blocks of size N = 6. Sealed, sequentially numbered, opaque envelopes with the study group allocation were opened by a nurse who was not involved in the study before the operation, and the PCA pump was prepared by the nurse and delivered to the anaesthesiologist. The PCA device was connected to the patient before the end of the operation. Nursing staff who were not involved in the study performed the pain assessment and follow-up postoperatively. Patients, anaesthesiologists, and outcome assessors were blinded to the treatment arm.

### Anaesthesia protocol

Routine monitoring was performed after entering the operating room, including electrocardiography, noninvasive blood pressure, and pulse oximetry. The haemodynamic index was monitored through radial artery pressure. Electrodes were applied to the patient’s forehead to monitor the bispectral index (BIS). Prior to anaesthesia induction, methylprednisolone 40 mg was given intravenously. After preoxygenation for 3 min, anaesthesia was induced with sufentanil 0.3–0.5 µg/kg, etomidate 0.2 mg/kg, rocuronium 0.6–0.9 mg/kg, and propofol 1 mg/kg, endotracheal intubation was performed 60 s after anaesthesia induction with a visual laryngoscope, and mechanical ventilation was initiated. Ultrasound-guided catheterization via the right internal jugular vein was performed. Next, the abdomen was scanned between the iliac crest and the costal margin at the mid-axillary line with a high-frequency linear ultrasound transducer to perform a transversus abdominis plane (TAP) block, and the skin over the anterolateral abdomen was cleaned and penetrated with a block needle using an in-plane technique. After visualizing the needle tip between the internal oblique and transversus abdominis muscles, 20 ml of 0.25% ropivacaine hydrochloride was injected after confirming negative aspiration for blood, and the two layers of muscles were separated by the local anaesthetic. The same technique was performed on the other side. For anaesthesia maintenance, remifentanil (0.05 ~ 0.2 µg/kg/min) and propofol (2 ~ 4 mg/kg/h) were given to the patient, sevoflurane was adjusted to keep the BIS between 45 and 60, and rocuronium (0.2 mg/kg) was added intermittently to maintain muscle relaxation until the pneumoperitoneum stopped. Patients were supplemented with sodium lactate Ringer’s solution according to goal-directed fluid therapy (GDFT) to maintain stroke volume variation (SVV) < 13%, ephedrine 5–6 mg or methoxamine 1 mg was administered to maintain blood pressure stability at ± 15% of the preoperative level.

The anaesthesia procedures were conducted by an attending anaesthesiologist, and the operations were performed by the same surgical team. Tropisetron 5 mg and hydromorphone hydrochloride 0.1 mg were given 30 min before the end of surgery, and the nurse connected the PCA to the patient. The anaesthetics were stopped 5 min before the end of the operation, and the endotracheal tube was removed after the recovery of consciousness and spontaneous breathing.

After the endotracheal tube was removed, patients were observed for 5 min and transferred to the postanaesthesia care unit (PACU) with SpO2 ≥ 95%. Patients were transferred to the inpatient ward when Aldrete’s score was a minimum of 9 points.

### Postoperative pain management

A standardized postoperative analgesic regimen was implemented. TAP block was performed on both sides after anaesthesia induction. Before the end of the surgery, hydromorphone hydrochloride 0.1 mg was injected intravenously, and the PCA device was connected to the patient. The PCA regimen was as follows: Group N1 received hydromorphone hydrochloride 0.15 mg/kg + nalmefene 2 µg/kg; Group N2 received hydromorphone hydrochloride 0.15 mg/kg + nalmefene 0.5 µg/kg; and the control group (Group C) received hydromorphone hydrochloride 0.15 mg/kg. All medications were diluted to 100 ml with normal saline and administered via continuous infusion at 2 ml/h (0.003 mg/kg/h), and a bolus dose of 1 ml (0.0015 mg/kg) with a lockout time of 15 min. If the postoperative NRS score was ≥ 4 or the patient was not satisfied after 3 bolus doses were administered continuously, flurbiprofen 50 mg was given intravenously. In addition, when the Ramsay score was over V, respiratory rate < 10/min or SpO_2_ < 90%, the analgesic pump was stopped. Then, the acute pain service (APS) staff reprogrammed the pump by decreasing the background dose and bolus dose of PCA by 25%. PCA was reconnected after the recovery of consciousness and respiration.

### Follow-up and data collection

Patient demographic data, surgical type (laparoscopic/hand-assisted laparoscopic), intraoperative blood loss, intraoperative transfusion volume, urine volume, and duration of surgery (defined as the time spent in the operating room in hours) were recorded.

The NRS score, PCA dosage and rescue analgesics were recorded at 1 h, 6 h, 12 h, 24 and 48 h after surgery. Pain intensity was evaluated using the numeric rating scale (NRS), with scores of 1–3 representing mild pain, scores of 4–6 representing moderate pain, and scores of 7–10 representing severe pain.

Drug-related adverse events, including nausea, vomiting, pruritus, respiratory depression [respiratory rate < 10 per min or pulse oxygen saturation (SpO2) < 90%], and the level of sedation, were assessed at each time point postoperatively. The level of sedation was classified using the Ramsay Sedation Scale: Grade I: patient is anxiety and/or restlessness; Grade II: patient is quiet, cooperative and accurate orientation; Grade III: patient responses to instructions only; Grade IV: patient exhibits brisk response to light glabellar tap or loud auditory stimulus; Grade V: patient exhibits a sluggish response to light glabellar tap or loud auditory stimulus; Grade VI: patient shows no response.

In addition, the time to postoperative exhaust, the time to drainage tube removal and the total length of hospital stay were also recorded from nursing records. Patient satisfaction was assessed at 48 h after surgery as follows: Grade I: very satisfied, Grade II: satisfied, Grade III: generally satisfied, Grade IV: dissatisfied, and Grade V: very dissatisfied.

The primary outcome was the NRS score at 12 h postoperatively. The secondary outcomes included the incidence of postoperative drug-related adverse events, the PCA dosage, the time to postoperative exhaust, the time to drainage tube removal, the total length of hospital stay, and patient satisfaction.

### Sample size

PASS 2022 was used to evaluate the sample size. The primary outcome was the pain score after surgery. Based on previous work [9], the mean of the numeric rating scale pain score at 6 h was 3.1 units. Under this assumption, it was determined that a sample size of n = 30 for each group would provide statistical power (two-tailed test, α = 0.05) of approximately 90% to detect a difference between groups of 0.9 units. Under the assumption that up to 10% of randomized subjects may be excluded for various reasons, a total sample size of N = 99 was used.

### Statistical analysis

SPSS Statistics (Version 21.0, IBM Corp, Armonk, NY, USA) was used for statistical analysis in this study. Descriptive statistics are used to present baseline characteristics for the three groups. Standard hypothesis tests (ANOVA, Kruskal‒Wallis test) were used to compare these baseline values among the three groups. Continuous variables were assessed for normality and presented as the mean (SD) or median (interquartile range) after checking the normality. Categorical variables were assessed using frequency tables and χ2 or Fisher’s exact test and are presented as frequencies and percentages. A P value less than 0.05 was considered statistically significant.

## Results

1. From June 2020 to September 2021, 127 patients were enrolled in the study, and 99 patients were randomly divided into three groups (n = 33). A total of 93 patients were included in the final analysis, with 31 in each group (Fig. 1). General demographic data and baseline characteristics were similar among the three groups, and no significant differences in the intraoperative conditions or surgical types were found among the three groups (Table 1).

2. Comparison of NRS scores of the three groups at different time points after the operation.

**Table 1:** Baseline Characteristics of the Patients in the Groups

	Group N1 (2 µg/kg) (n = 31)	Group N2 (0.5 µg/kg) (n = 31)	Group C (n = 31)	F value	χ^2^ value	P value
Sex, female/male	17/14	14/17	12/19		1.644	0.44
Age (year, $\bar x \pm s$)	61 ± 12	59 ± 12	61 ± 12	0.169		0.845
Height (cm, $\bar x \pm s$)	164 ± 8	165 ± 9	167 ± 6	1.542		0.22
Weight (kg, $\bar x \pm s$)	62 ± 13	66 ± 12	66 ± 11	1.233		0.296
ASA grade (I/II/III)	0/22/9	0/26/5	0/24/7		1.476	0.478
Transfusion volume (ml, $\bar x \pm s$)	2337 ± 717	2114 ± 509	2484 ± 579	2.900		0.06
Blood loss (ml, $\bar x \pm s$)	141 ± 138	141 ± 104	148 ± 122	0.04		0.961
Urine output (ml, $\bar x \pm s$)	309 ± 264	247 ± 256	349 ± 323	1.026		0.363
Duration of surgery (h, $\bar x \pm s$)	3.91 ± 0.76	3.70 ± 0.84	4.18 ± 0.80	2.866		0.062
Surgical type (laparoscopic/hand-assisted laparoscopic)	22/9	22/9	27/4		2.977	0.226

The differences in NRS scores were compared at different time points among the three groups. The NRS scores of Group N1 (2 µg/kg) were significantly lower than those of Group C (P = 0.025), but no difference was found between group N2 and group C (P > 0.05).

**Fig. 2:**
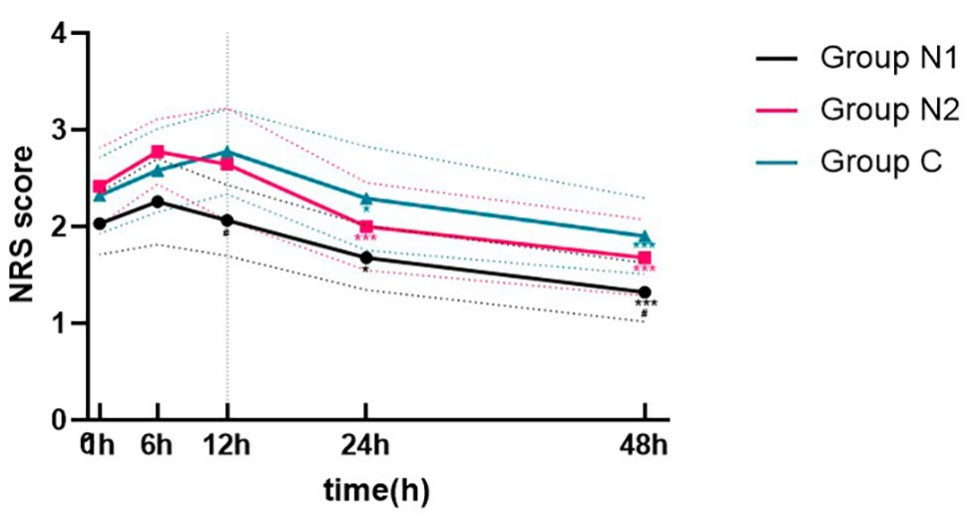
Comparison of NRS scores of the three groups at different time points after the operation. Figure Repeated-measures ANOVA was used to compare the differences in NRS scores among the three groups at different time points. ^#^P: Compared with the control group (Group C), the NRS scores of Group N1 (2 µg/kg) were significantly lower at 12 and 48 h postoperatively (P < 0.05). ^*^P: Compared with the 12 h postoperative time point, the NRS scores at 24 h were lower in Group N1 and Group C (P < 0.05). ^***^P: Compared with the 12 h postoperative time point, the NRS scores were significantly lower at 24 h in Group N2 and at 48 h in all three groups (P < 0.001)

As shown in Fig. 2, among the three groups, the NRS scores of Group N1 (2 µg/kg) were significantly lower than those of Group C at 12 h (P = 0.01) and 48 h (P = 0.01) postoperatively. However, there was no significant difference between Group N2 and Group C, and no difference was found between Group N1 and Group N2.

In addition, compared with 12 h postoperatively, the NRS scores were lower at 24 h postoperatively in Group N1 and Group C (P < 0.05) and significantly lower at 48 h postoperatively in all three groups (P < 0.001) (Fig. 2).

**Fig. 3:**
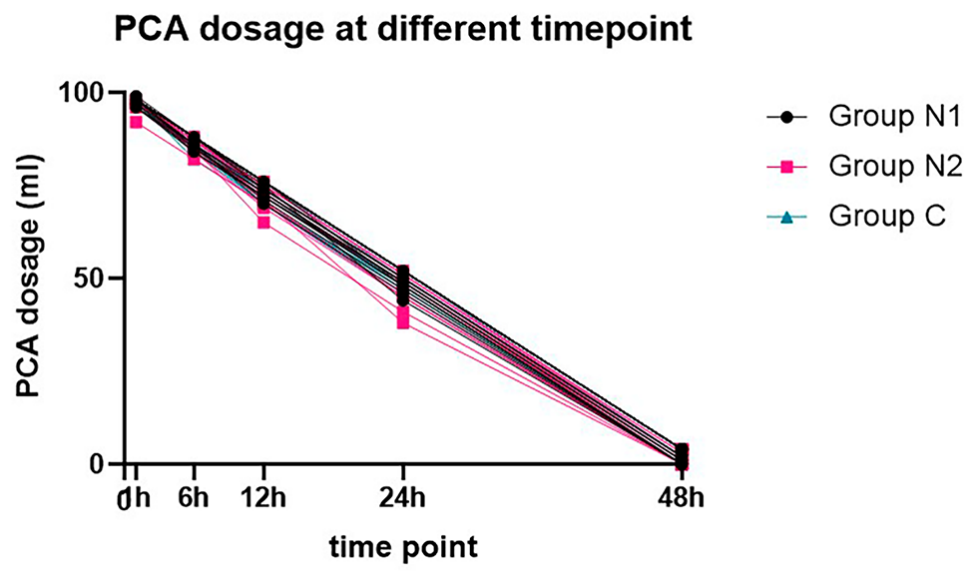
PCA dosage at different timepoints. There was no difference among the three groups in total analgesic consumption at any time point postoperatively

However, as shown in Fig. 3, there was no difference in total analgesic consumption at any time point among the three groups.

3. Comparison of postoperative adverse events.

There was no significant difference in the incidence of either nausea (P = 0.353) or vomiting (P = 0.875) among the three groups. No patients needed medication to treat severe nausea and vomiting in the three groups. There was a significant difference in the incidence of pruritus among the three groups, with 0 (0%) patients in Groups N1 and N2 and 4 (2.6%) patients in Group C (P = 0.036) (Table 2). However, no patients needed medical intervention for severe pruritus in the control group. There were no episodes of respiratory depression, indicated by a respiratory rate less than 10 breaths/min or a desaturation event with SpO_2_ less than 90%, in any patients in the three groups.

**Table 2:** Incidence of postoperative adverse reactions in the three groups. There were significant differences in the incidence of pruritus among the three groups

	Group N1 (2 µg/kg) (n = 31)	Group N2 (0.5 µg/kg) (n = 31)	Group C (n = 31)	χ2 value	Fisher exact test	P value
Nausea %	7(4.5)	13(8.4)	12(7.7)	2.081		0.353
Vomiting %	3(1.9)	1(0.6)	2(1.3)		1.044	0.875
Pruritus %	0(0)	0(0)	4(2.6)		5.706	**0.036**

4. The exhaustion time (P = 0.372), drainage tube extubation time (P = 0.758) and total length of hospital stay (P = 0.938) did not differ among the three groups. There were no differences in patient satisfaction among the three groups (P = 0.132), and no patients were unsatisfied with this PCA regimen (Table 3).

**Table 3:** Comparison of outcomes and patient satisfaction among the three groups

	Group N1 (2 µg/kg) (n = 31)	Group N2 (0.5 µg/kg) (n = 31)	Group C (n = 31)	F value	χ^2^ value	P value
Exhausting time (d, $\bar x \pm s$)	2.68 ± 1.33	2.26 ± 0.86	2.65 ± 1.60	1.001		0.372
Drainage tube extubation time (d, $\bar x \pm s$)	8.19 ± 4.45	7.87 ± 3.15	7.54 ± 2.26	0.278		0.758
Total length of hospital stay (d, $\bar x \pm s$)	18.03 ± 5.46	17.54 ± 5.58	17.83 ± 5.02	0.064		0.938
Patient satisfaction (1/2/3)	9/21/1	2/27/2	7/24/0		7.083	0.132

## Discussion

The results of this study show that nalmefene at a dosage of 2 µg/kg can enhance the analgesic efficacy of hydromorphone hydrochloride and reduce the occurrence of postoperative pruritus without affecting the postoperative outcome of patients. For the first time, our study used nalmefene combined with hydromorphone hydrochloride in a multimodal analgesia regimen in colorectal surgery.

1. Application of hydromorphone hydrochloride in postoperative analgesia.

Hydromorphone hydrochloride is currently widely used in postoperative analgesia [10]. Yang et al. reported that hydromorphone IV-PCA had an effective postoperative analgesic effect for patients undergoing radical colorectal surgery [4]. In our study, a continuous infusion of hydromorphone at 0.003 mg/kg/h was given to the patients, and the results showed that the analgesic regimen was considered to be effective for the management of postoperative analgesia, which was consistent with the findings in the literature. A previous study on postoperative analgesia with hydromorphone hydrochloride in patients undergoing single-port thoracoscopy revealed that the PCA mode of a bolus dose plus background infusion was more effective than that of a single additional dose alone for pain control [11]. In our study, a background dosage of hydromorphone hydrochloride at 0.003 mg/kg/h combined with a single additional dose of 0.0015 mg/kg was used as the PCA regimen, there was no significant difference in the frequency of PCA among the groups, and no rescue analgesic drugs were used in patients postoperatively.

2. Nalmefene dosage.

A previous study on postoperative analgesia for lower abdominal surgery found that 15 or 25 µg nalmefene via intravenous administration at the end of the operation could reduce the application of antiemetic and antipruritic drugs within 24 h after the operation [8]. At present, the duration of postoperative analgesia in our hospital is 48 to 72 h. Considering that the half-life of nalmefene is 10.8 h and that a single dose is not enough to provide the analgesic effect for two days postoperatively, low-doses of nalmefene combined with hydromorphone hydrochloride was infused continuously for two days. Based on a literature review, the dosage of naloxone combined with opioids for the analgesic pump was 0.25 µg/kg/h, and the 48-hour dose was approximately 12 µg/kg [12, 13]. Therefore, in our study, considering that the affinity of nalmefene to µ-receptors is approximately 4 times that of naloxone and to ensure that the total daily dose of nalmefene was not greater than 1 µg/kg to avoid affecting the analgesic effect of opioids, we set the total dose of nalmefene as 2 µg/kg in Group N1. Moreover, as the affinity of nalmefene for the κ receptor is approximately 28 times that of naloxone, the total dose of nalmefene in Group N2 was 0.5 µg/kg [14].

3. Effect of nalmefene on analgesia.

In this study, the NRS scores at 12 and 48 h postoperatively were significantly lower in the 2 µg/kg nalmefene group (Group N1) than in the control group, which indicated that 2 µg/kg nalmefene could enhance the postoperative analgesic effect of hydromorphone hydrochloride. Low-dose opioid receptor antagonists can enhance the analgesic effect of opioids by blocking the excitatory opioid receptor signal transduction of dorsal root ganglion neurons, shortening the duration of the action potential and inhibiting the release of enkephalin and the microglia-mediated inflammatory response [15, 16]. Naloxone is a classic opioid receptor antagonist, while nalmefene is a new generation drug, and its affinity for the µ receptor is four times that of naloxone. Studies have found that [17] naloxone infusion at 0.25 µg/kg/h not only attenuates side effects but also reduces postoperative opioid requirements, which was consistent with the findings in our research. Furthermore, Cheung found that naloxone tended to reduce opioid requirements in the postnaloxone period [18]. However, Cepeda found that adding 6 µg/ml naloxone in morphine PCA for continuous infusion did not decrease opioid requirements [19], which revealed that the appropriate dose of naloxone, which was also for nalmefene, was not determined. In the present study, there was no significant difference in the NRS scores at 1 and 6 h postoperatively when compared with those at 12 h within all three groups, and the NRS scores were in the mild range. In addition, the PCA dosage did not show statistically significant differences among the three groups, which might be related to the application of TAP block in multimodal analgesia. Considering that the surgical incision would cause severe pain postoperatively, we performed TAP block for the patients. Sun found that TAP block with 0.375% ropivacaine could reduce the postoperative pain score of colorectal cancer patients within 12 h postoperatively, which was consistent with the results of this study [20]. In addition, our research provides a possible effective dose for the clinical application of nalmefene.

4. Effects of nalmefene on postoperative adverse events and outcomes.

According to the additional results of our study, the incidence of postoperative pruritus in the two nalmefene groups was lower than that in the control group, but there was no significant difference in the incidence of nausea and vomiting, which was consistent with other reports. Hydromorphone hydrochloride activates the µ, κ and δ receptors at the spinal cord level, but it also inhibits the activity of interneurons simultaneously, which consequently induces pruritus and other adverse events, and low-dose opioid receptor antagonists can reduce the occurrence of opioid-related adverse events [21]. Furthermore, the incidence of pruritus in the two nalmefene groups (0%) was significantly lower than that in the control group (2.6%), which had obvious clinical significance. However, this may be attributed to the relatively smaller sample size, and further study is necessary on this issue in the future. Considering that sugammadex or neostigmine may cause nausea and vomiting, patients were not given muscle relaxant antagonists. In addition, all patients were given low-dose glucocorticoids during the perioperative period and antiemetic drugs for ethical reasons before the end of the operation, which led to a reduced incidence of postoperative nausea and vomiting. Hence, there was no significant difference in such adverse reactions among the three groups [22].

In addition, our research group could not verify the effect of nalmefene on gastrointestinal function after colorectal surgery. Nevertheless, in prior research, naloxone and morphine were injected into the epidural space of rabbits, and the incidence of constipation in the naloxone and morphine groups was lower than that in the morphine group, showing a faster recovery of gastrointestinal motility. The occurrence of constipation in rabbits might be related to the decreased expression of interstitial cells of Cajal (ICCs) in the proximal colon; importantly, the exogenous use of naloxone can promote a faster recovery of gastrointestinal motility, as it can reverse the changes in ICCs and restore the ICC count [23]. However, few studies have focused on the gastrointestinal motility of nalmefene, and future studies could be carried out to further investigate this subject.

Our study has a few limitations. Notably, we performed TAP block after induction of general anaesthesia, so the exact blocking area was not tested. However, all procedures were conducted by attending anaesthesiologists and guided by an ultrasound transducer to ensure that the anaesthetic was injected between the correct layers, which was intended to minimize the individual differences in the blocking area. Second, although using TAP block for abdominal surgery is appropriate in clinical practice, it potentially limits the ability to observe differences in pain scores among groups. Similarly, the glucocorticoids and combined intravenous-volatile anaesthesia methods might also impact the occurrence of nausea and vomiting. Additionally, our research was designed as a single-centre study focusing on postoperative analgesia in patients undergoing colorectal cancer surgery. In the future, multicentre studies with larger samples are needed to verify the application of nalmefene in other operations.

## Conclusions

In conclusion, the findings of our study suggest that, during postoperative analgesia with hydromorphone hydrochloride, the addition of nalmefene at a dose of 2 µg/kg can enhance the postoperative analgesic effect of the drug and reduce the occurrence of postoperative pruritus.

## Data Availability

All data generated or analysed during this study are included within the article and related files.

## Abbreviations

PCA: Patient-controlled analgesia
TAP block: Transversus abdominis plane block
ASA: American Society of Anaesthesiologists
5-HT3: 5-hydroxytryptamine 3
GDFT: Goal-directed fluid therapy
SVV: Stroke volume variation
NRS: Numeric rating scale
TCI-PCA: PCA with target-controlled infusion
ICCs: Interstitial cells of Cajal
